# Compassionate-use of autologous, bone marrow–derived mesenchymal stromal cells in a chronic anoxic encephalopathy: a case report

**DOI:** 10.3389/fncel.2026.1822330

**Published:** 2026-07-27

**Authors:** Guilherme Lepski, Valeria Modestto, Walker Jeveaux, Daniel Navarro da Rocha, Roberto Dalto Fanganiello, Jose Ricardo Muniz Ferreira, Analia Arevalo

**Affiliations:** 1 Laboratory of Experimental Surgery, LIM26, Medical School, University of São Paulo, Sao Paulo, Brazil; 2 Libertare Institute – Neurology, Neurophysiology and Education, São Paulo, Brazil; 3 R-Crio Criogenia S.A., Campinas, Brazil; 4 Faculty of Science and Engineering, Université Laval, Quebec, QC, Canada

**Keywords:** mesenchymal stem cells, cell therapy, transplantation, neuroregeneration, neurologic diseases

## Abstract

**Introduction:**

Severe hypoxic-ischemic brain injury may evolve into prolonged disorders of consciousness, for which disease-modifying interventions remain a challenge. Mesenchymal stromal/stem cells (MSCs) are being investigated in neurological disorders predominantly through immunomodulatory and trophic paracrine mechanisms rather than neuronal replacement.

**Methods:**

We describe a young adult medicine student with chronic post-anoxic encephalopathy managed under national regulatory oversight for compassionate use of an advanced cellular therapy. Autologous bone marrow-derived MSCs were harvested and expanded under GMP-oriented controls with extensive biosecurity and release testing. Quantitative EEG and source localization with low-resolution electromagnetic tomography (LORETA) were performed before transplantation and 4 months thereafter.

**Results:**

Four months after intrathecal administration, subtle but consistent behavioral changes were observed, including more synchronized affective responses, greater environmental engagement, and the first reproducible binary interaction via eye blinking. Retrospective review of available video documentation showed an increase in the Coma Recovery Scale-Revised (CRS-R) score from 8 before treatment to 11 after treatment, with one-point improvements in auditory function, visual function, and arousal. Modified Ashworth Scale spasticity scores improved by 1 in some muscle groups. No adverse events were registered. Longitudinal quantitative EEG demonstrated persistent global slowing compatible with severe diffuse injury, with qualitative emergence of faster activity, high alpha (10–12 Hz) and low beta (12–18 Hz) in bilateral fronto-temporal areas. LORETA current-density maps showed a more anterior distribution of estimated generators toward fronto-limbic regions.

**Discussion:**

This case illustrates the feasibility and short-term safety of regulated autologous MSC delivered intrathecally in chronic post-anoxic encephalopathy, while emphasizing that therapeutic efficacy cannot be inferred from a single uncontrolled observation.

## Introduction

Mesenchymal stromal/stem cells (MSCs) have moved from experimental “cell replacement” concepts toward clinically testable biologics for neurological disease, largely on the basis of paracrine immunomodulatory and trophic mechanisms that may reshape neuroinflammation, vascular dysfunction, and maladaptive glial responses rather than directly generating new neurons. In humans, this has translated into a growing body of case series and early- to mid-phase clinical trials in which MSC products are administered systemically (most commonly intravenous) or delivered more directly to the central nervous system (intrathecal and, less often, intraspinal/perilesional), with outcome measures spanning impairment (e.g., AIS/ASIA, EDSS, NIHSS), function (e.g., FIM, walking metrics), patient-reported quality of life, and imaging/biomarker readouts. Recent studies are increasingly designed as randomized controlled trials (RCTs), include predefined safety stopping rules, and report product characterization and release testing consistent with contemporary “biosecurity” expectations for cell-based interventions—namely donor screening (for allogeneic products), aseptic processing, sterility/endotoxin/mycoplasma testing, and control of culture-related genetic instability through limited passage and lot release criteria (Nguyen et al., 2025; Schiess et al., 2025; Harris et al., 2024; Bydon et al., 2024).

Across neurological indications, the dominant clinical signal to date is that MSC administration—when performed under controlled trial conditions—has shown an acceptable short-term safety profile, while definitive efficacy remains indication- and endpoint-dependent and often modest. Safety events reported in modern trials tend to cluster around (i) procedure/route-related events (e.g., post-lumbar puncture headache, transient back pain, meningeal symptoms after intrathecal dosing), (ii) infusion-related reactions (e.g., transient fever, chills, fatigue), and (iii) disease-related events expected in fragile neurologic populations. Importantly, several recent trials and longer follow-ups have not identified clear signals of ectopic tissue formation, neoplasia, or sustained alloimmune toxicity attributable to MSC products, although these risks remain a central biosecurity concern that motivates stringent manufacturing controls and long-term surveillance in trial protocols (Bydon et al., 2024; Awidi et al., 2024; Nguyen et al., 2025; Schiess et al., 2025; Akhlaghpasand et al., 2025a,b; Amanat et al., 2021; Honmou et al., 2021; Kaplan et al., 2025). The relative contribution of product-intrinsic factors (cell source, donor variability, culture conditions, potency) versus delivery strategy (route, dose, schedule) has become a key interpretive challenge, and recent trials increasingly emphasize potency assays and batch consistency as plausible determinants of clinical signal detection (Schiess et al., 2025).

Only a limited number of reports have specifically addressed MSC-based interventions in chronic severe brain injury (Kawabori et al., 2021; Okonkwo et al., 2024), disorders of consciousness, or hypoxic-anoxic encephalopathy (Wada et al., 2025; Xie et al., 2016). This scarcity naturally restricts indication-specific conclusions and requires cautious interpretation. Available reports in chronic severe brain injury/disorders of consciousness have mainly evaluated feasibility and short-term safety, including systemically administered autologous MSC products expanded under defined conditions, while efficacy remains unproven and requires controlled trials (Hirota et al., 2024; Yamaki et al., 2024).

The rationale for the present compassionate-use case was therefore not to infer efficacy but to document regulated manufacturing, biosecurity controls, route selection, adverse-event surveillance, and exploratory clinical and qEEG follow-up. At this stage of development, especially for chronic post-anoxic encephalopathy, the central scientific question remains whether autologous MSCs can be manufactured, released, and administered safely under controlled conditions. Autologous bone marrow-derived MSCs were selected to minimize immunological compatibility concerns and donor-related variability. The intrathecal route was chosen because chronic post-anoxic encephalopathy primarily involves CNS dysfunction in the setting of an intact or reconstituted blood-brain barrier, which may limit the access of intravenously administered cells or cell-derived paracrine mediators to the CNS compartment. Intrathecal administration was therefore considered a more direct route to the cerebrospinal fluid space while avoiding the higher procedural risk of intraparenchymal delivery.

## Case description (baseline and clinical context)

LMC is a female, 32-year-old medical student who developed a severe disorder of consciousness after a perioperative hypoxic–ischemic event. She underwent corrective orthognathic surgery in March 2023 and presented a post-anesthetic cardiac arrest lasting ∼20 min, requiring cardiopulmonary resuscitation. The subsequent ICU course included aspiration bronchopneumonia and urinary tract infection, followed by prolonged hospitalization and discharge approximately 1 year later. At the time of institutional evaluation for advanced cell therapy (Feb/2025), the working diagnosis was severe chronic hypoxic–ischemic encephalopathy with persistent impairment of consciousness and spastic quadriparesis. Neuroimaging was described as lacking large territorial ischemic lesions, encephalomalacia, or hematoma, but with FLAIR hyperintensity involving basal ganglia and fronto-parieto-occipital cortices compatible with anoxic encephalopathy.

Upon neurological examination (Feb/2025), the patient exhibited tracheostomy and gastrostomy, spontaneous breathing in room air, and bilateral spastic pyramidal involvement, clinically expressed as spastic tetraparesis affecting all four limbs, without demonstrable voluntary limb motor activity; automatic antalgic movements were observed. No consistent, reproducible communication could be established at that time, including unsuccessful attempts to implement an eye-blink strategy. Visual contact was inconsistent, with intermittent gaze engagement mainly when called by her parents and otherwise a vacant stare.

Before MSC administration, the patient had been receiving continuous intensive multidisciplinary rehabilitation 7 days per week, approximately 3 h per day, including conventional physical and speech-language therapy, oral sensorimotor stimulation, receptive language-directed therapy, music therapy, transcutaneous electrical nerve stimulation, functional electrical stimulation, photobiomodulation, and non-invasive neuromodulation with transcranial direct current stimulation (tDCS). Two tDCS protocols were used in alternation, one targeting frontal and temporal regions, and another targeting frontal and cerebellar regions. Regular medications included baclofen, duloxetine, pregabalin, nitrofurantoin, and amantadine. The long interval between injury and MSC administration, together with limited neurological change despite sustained rehabilitation, makes spontaneous late recovery less likely, although delayed fluctuation or rehabilitation-related effects cannot be completely excluded in a single uncontrolled case.

A quantitative EEG performed on 29/08/2025 described very low global electrical power with diffuse slowing, persistent severe network disorganization, in the context of severe diffuse hypoxic injury.

## Ethics and regulatory oversight

This intervention was conducted under the oversight of the Brazilian National Health Surveillance Agency (ANVISA), within the framework applicable to the compassionate/non-routine use of advanced therapy products (process no. 25351.056687/2025-45). According to Brazilian regulations, a medical-technical report containing the scientific and medical justification for the procedure, a detailed description of the GMP-compliant cell-production protocol and documented legal consent must be submitted to the agency. In addition, a follow-up report is mandatory after 6 months, or earlier in case of any adverse event.

The patient’s legal representatives (parents) executed a written informed consent form for autologous mesenchymal stromal/stem cell (MSC)-based advanced therapy after discussion of the exploratory nature of the intervention.

## Methods

Autologous MSCs were obtained by bone marrow aspiration through iliac crest puncture under local anesthesia and aseptic conditions.

### Manufacturing and expansion under GMP-oriented controls

**FIGURE 1:**
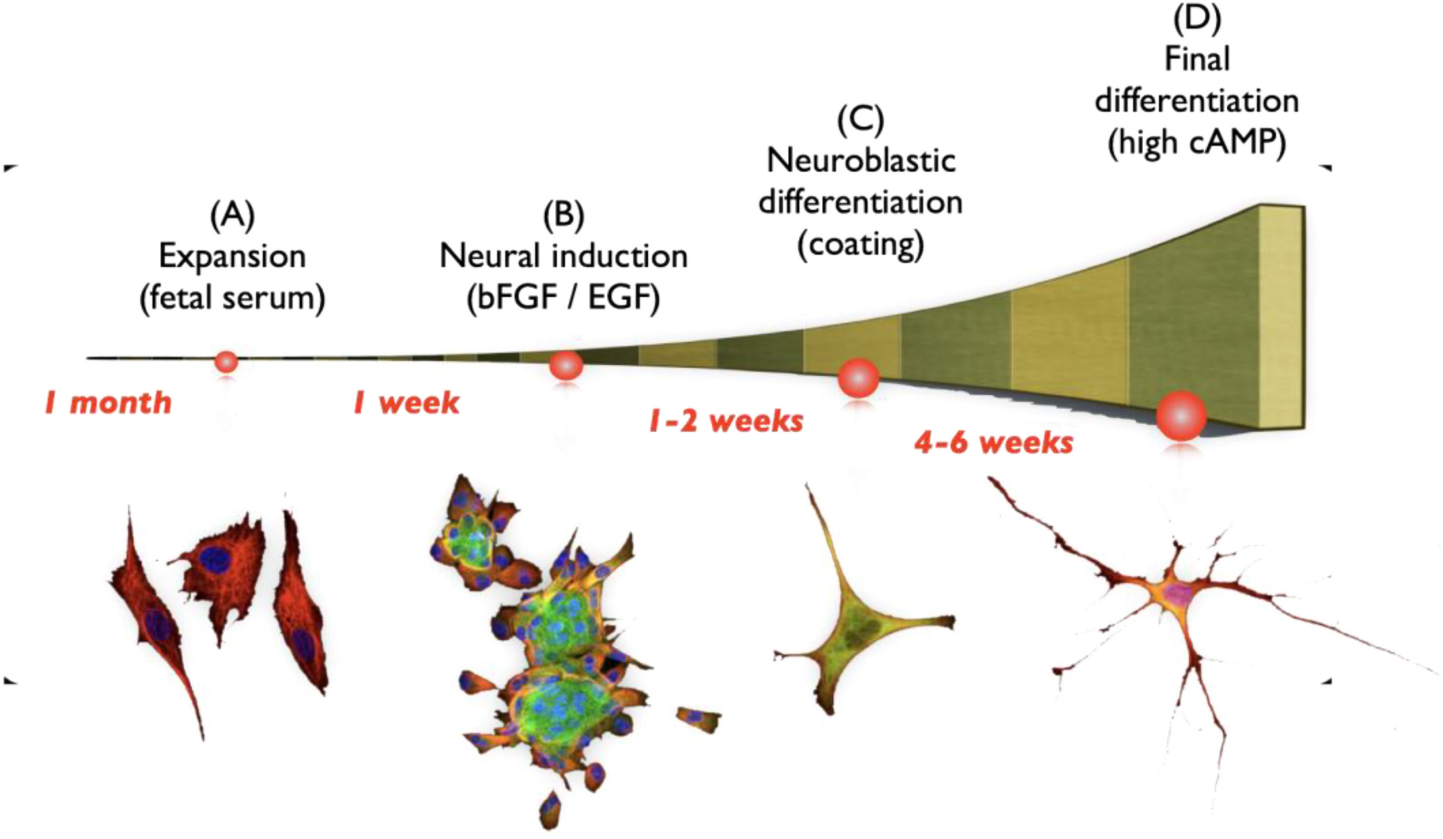
Experimental background for neuronal induction of mesenchymal stromal/stem cells *in vitro*. **(A)** Expansion phase in medium containing 10% fetal serum, lasting at least 1 month; **(B)** neural induction phase, during which cells are cultivated in medium containing bFGF and EGF; **(C)** neuroblast-like differentiation after transfer to flasks coated with poly-L-ornithine or fibronectin; and **(D)** final differentiation after culture in medium enriched with cAMP. The Figure summarizes previous experimental work from our group and the culture rationale; it is not presented as evidence of neuronal replacement in the treated patient.

Cell processing and expansion were performed in a dedicated manufacturing environment (R-CRIO Criogenia S/A, Campinas, Brazil). Culture duration ranged from 3 to 5 months. Cells were expanded through 6 to 10 passages, with passaging performed after achievement of approximately 50% confluence. The manufacturing process was as follows (Figure 1):

- Primary processing with density-gradient separation (BioColl, Sigma-Aldrich, USA) to isolate mononuclear cells, followed by culture in Human Mesenchymal-XF Expansion Medium (Millipore, USA) supplemented with human serum (Sigma-Aldrich, USA), 2 mM L-glutamine (Sigma-Aldrich, USA), ascorbic acid (Sigma-Aldrich, USA), and 100 U/mL and 100 μg/mL, respectively penicillin/streptomycin (Sigma-Aldrich, USA) and serial monitoring of adherence, fusiform morphology, and homogeneous growth, with media changes and confluency-based passaging (70%–90% confluence).
- After isolation and expansion, cells were exposed for 48 h before infusion to a serum-free neuronal induction medium containing xenofree mesenchymal expansion medium (Millipore, USA), Neurobasal medium, CTS B-27 Supplement XenoFree (50X, Thermo Fisher, Gibco), penicillin/streptomycin (Nova Biotecnologia), and the growth factors EGF and bFGF (20 ng/mL each, ProteinTech). This induction step was based on previous experimental work from our group and is not presented as evidence that neuronal replacement occurred *in vivo*.
- Cryopreservation in a matrix containing human serum (Sigma-Aldrich, USA) and DMSO (Origen Biomedical, USA), with controlled-rate freezing (−1 °C/min) to below −150 °C, and segregation of supernatant for quality control testing.

### Biosecurity and product release testing

Biosecurity measures comprised: (i) donor/patient laboratory screening for transfusion-transmissible infections consistent with RDC 836/2023 (including Chagas disease, HBV markers, HCV, HIV with p24, HTLV I/II, malaria in relevant exposure settings, and syphilis), and (ii) microbiological release assays on the manufactured product, including sterility, endotoxin, and mycoplasma testing. Product identity was assessed by flow cytometry, with expected MSC-positive markers (CD105, CD73, CD90) and low or absent expression of hematopoietic/activation markers (CD34, CD44, CD45, and HLA-DR). Viability was assessed using trypan blue exclusion and was above 97% at the time of infusion.

### Final formulation, handling, and route of administration

The final product consisted of 1.0 × 10^6 autologous MSCs in 1 mL, suspended in PBS with calcium and magnesium and 80 mg/dL glucose, provided in a sterile vial for injection. The dose was intentionally conservative because this was an exploratory compassionate-use intervention. The rationale prioritized tolerability and biosecurity, considering the approximate adult cerebrospinal fluid volume and the diagnostic relevance of CSF pleocytosis after intrathecal administration. The selected dose was intended to remain below a cell burden that could complicate interpretation of post-infusion fever or CSF inflammatory changes as reactive meningitis.

Administration was performed as a single intrathecal infusion by lumbar puncture on September 17, 2025. A single administration was selected to minimize cumulative procedural and biological risk. Although repeated administration may theoretically produce additive effects, there is currently no definitive evidence proving superiority of repeated dosing over a single infusion in this specific clinical context.

### Clinical assessment

The patient was examined before the intervention and again 4 months thereafter. Blinded video documentation was presented to an independent neurologist, who retrospectively determined the scores on the Coma Recovery Scale–Revised (Bodien et al., 2016; Giacino et al., 2004; Weaver et al., 2024), modified Rankin Scale, Extended Glasgow Outcome Scale, and Modified Ashworth scales.

Adverse events were actively monitored according to the standardized pharmacovigilance and safety-surveillance framework required by ANVISA for advanced therapy products, using the Common Terminology Criteria for Adverse Events (CTCAE) version 6.0 for classification and severity grading (National Cancer Institute, 2025).

### Electrophysiological recording and quantitative analysis

Quantitative EEG (qEEG) was recorded using a standard international 10–20 electrode placement system (Niedermeyer and da Silva, 2005) with a Mitsar EEG-201 amplifier and processed in WinEEG, including low-resolution electromagnetic tomography/standardized low-resolution electromagnetic tomography (LORETA/sLORETA) source-localization modules (Pascual-Marqui et al., 1994; Pascual-Marqui, 2002). Electrode impedances were maintained between 5 and 10 kOhm. Data were acquired at a sampling rate of 1 kHz with average reference. Signals were band-pass filtered from 0.5 to 50 Hz. The recording was obtained under vigilance, with acquisition judged as good technical quality, and included eyes-open and eyes-closed conditions. Independent component analysis was applied as part of the preprocessing pipeline, and artifact-contaminated segments were excluded before spectral analysis. Only technically acceptable fragments were retained. For spectral mapping, artifact-controlled fragments were segmented and quantified for each behavioral condition. In the pre-treatment examination, the eyes-closed segment had 470.04 s duration (offset 2.00 s) with 227 epochs, and the eyes-open segment had 375.58 s duration (offset 1.72 s) with 143 epochs. In the follow-up examination, the eyes-closed segment had 377.28 s duration, comprising 54 epochs, and the eyes-open segment lasted 375.58 s with 14 artifact-free epochs (this exam had a higher movement- and engagement-related artifacts burden). The qEEG analysis was longitudinal and within-patient, rather than a normative or group-based statistical comparison. Inferential statistical analysis was not performed because of the exploratory single-case design.

## Main results (early clinical evolution)

### Pre-infusion functional state

Immediately prior to the intervention, the patient exhibited no reproducible emotional interaction and no consistent eye-contact-based communication, with absent reliable command-following and no functional voluntary motor output, consistent with chronic severe disorder of consciousness and spastic tetraparesis. Retrospective baseline scoring showed CRS-R 8, mRS 5, GOS-E 3, and Modified Ashworth Scale grade 3 in the major muscle groups of all 4 limbs.

### Four-months follow-up (post-infusion)

At the structured follow-up visit approximately 4 months after intrathecal MSC infusion, no adverse events were recorded, including no procedure-related complications, infectious events, inflammatory reactions, neurological deterioration, ectopic tissue formation, neoplastic complications, or systemic adverse events. The clinical evolution was characterized by subtle but consistent behavioral changes, reported by the family and caregivers and also video-documented by the authors for scoring:

- Emergence of more synchronized, context-congruent emotional reactions, including smiling and crying;
- Greater orientation toward family members and therapists, with increased responsiveness to auditory and visual stimulation;
- Increased satisfaction with musical stimuli
- Development of a limited but functionally meaningful and reproducible eye-blink–based contact/communication (not present before treatment);
- Less pain behavior and greater tolerance during mobilization; more specifically, Modified Ashworth Scale scores reduced from 3 (in all four limbs) to 2 in some muscle groups (like biceps, triceps, hamstrings and anterior tibial muscles).
- Retrospective post-treatment scoring showed CRS-R 11, reflecting one-point gains in auditory function, visual function, and arousal, respectively. The mRS remained 5, the GOS-E remained 3.

### Electrophysiology

**FIGURE 2:**
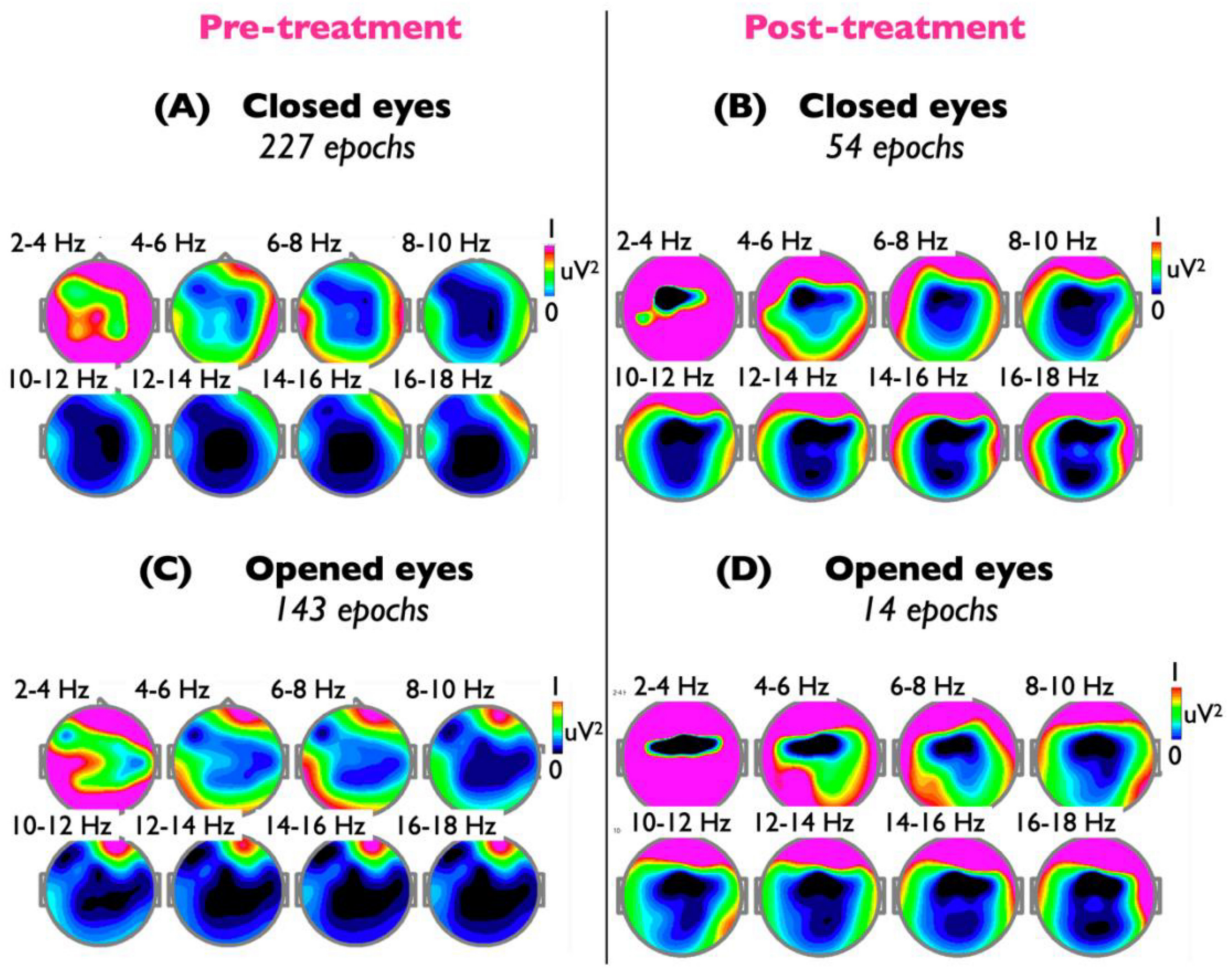
Quantitative EEG (qEEG) results: scalp band-power maps and spectral patterns before and after treatment. On the left **(A,C)**, pre-treatment recordings; on the right side **(B,D)**, 4 months follow-up recordings. Scalp topographies of delta (2–4 Hz), low theta (4–6 Hz), and slow-alpha (6–8 Hz), band power under eyes-closed **(A)** and eyes-open **(C)** conditions in the pre-treatment session. Delta activity is diffuse and dominant in both states, while slow-alpha remains weak and non-posteriorly dominant; the visual similarity between eyes-closed and eyes-open maps is consistent with limited state-dependent modulation. **(B,D)** Follow-up with eyes-closed and opened power spectra and peak-frequency maps displayed more pronounced faster activity, high alpha 10–12 Hz and low beta 12–18 Hz in fronto-temporal areas bilaterally. The figure is presented as a descriptive longitudinal comparison; no inferential statistical analysis or normative comparison was performed.

At follow-up, the electrophysiological profile was dominated by diffuse background slowing with predominant delta activity (2–4 Hz) distributed broadly across cortical regions, accompanied by markedly reduced absolute power (generally < 5 microV). Against this globally slowed, low-amplitude background, an incipient slow alpha component (7.08–8.30 Hz) was identified, topographically localized to bilateral temporal regions with greater expression in the left hemisphere. Theta activity (4–7 Hz) was also present over frontal and temporal regions, remaining low amplitude. State modulation was limited, with minimal condition-dependent spectral change between eyes-open and eyes-closed recordings (Figure 2).

Longitudinally, the dominant phenotype remained diffuse delta/theta predominance with low global power, with qualitative emergence of more evident faster activity, including high alpha (10–12 Hz) and low beta (12–18 Hz) in bilateral fronto-temporal areas.

**FIGURE 3:**
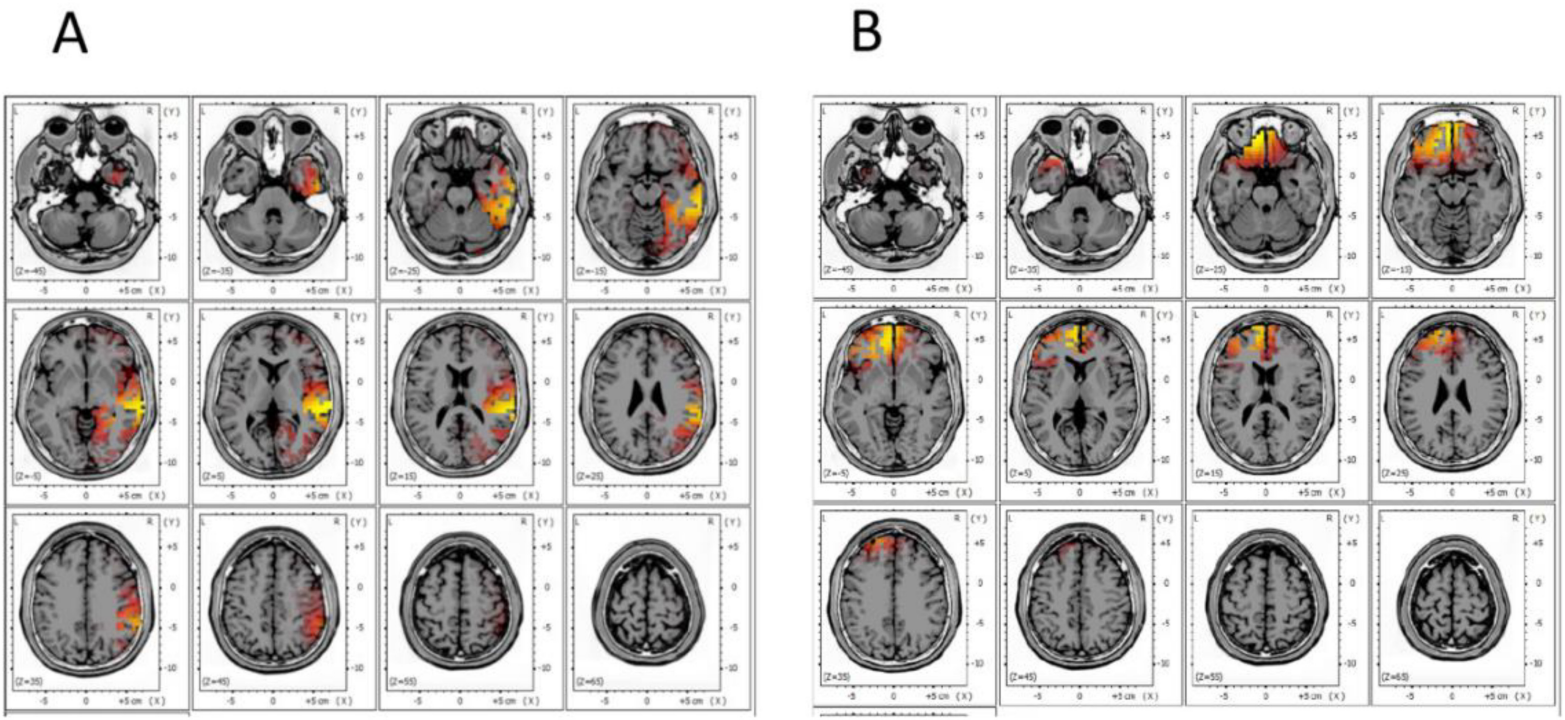
Source estimation of slow-wave activity before and after treatment. **(A)** Pre-treatment (baseline): LORETA/sLORETA current-density maps show that the dominant slow activity was mainly generated in temporal and temporoparietal cortices, encompassing Brodmann areas (BA) 41/42, 21/22, and 40. **(B)** Post-treatment: The main generators shifted anteriorly toward frontal and fronto-limbic regions, including BA 10, 11, 32, 47, and 25. Warmer colors indicate higher estimated current density on a relative scale. Because LORETA/sLORETA is a low-resolution source-estimation method, these maps should be interpreted descriptively and not as precise anatomical localization or functional confirmation of network recovery.

Pre-treatment LORETA/sLORETA (Figure 3) current-density maps estimated dominant slow activity mainly in temporal and temporoparietal cortices, encompassing Brodmann areas (BA) 41/42, 21/22, and 40, whereas the post-treatment examination showed a more anterior distribution of estimated generators toward frontal and fronto-limbic regions, including BA 10, 11, 32, 47, and 25. These source-localization findings are descriptive and should not be interpreted as precise anatomical localizations.

## Discussion

The longitudinal qEEG profile remains dominated by severe global dysfunction—very low absolute power with diffuse slow activity—yet the follow-up recording shows a subtle change in how the residual oscillatory activity is organized. At baseline, the record was essentially constrained to diffuse delta–theta activity with only a focal, right-frontal alpha-like component, whereas follow-up preserved the delta-dominant background but added a weak, bilaterally expressed slow-alpha component in temporal regions together with more evident theta activity over frontal and temporal cortices. Nevertheless, these changes should be interpreted cautiously, since they do not demonstrate normalization or significant functional recovery, but instead a small shift from a largely undifferentiated slow background toward a modestly more structured residual rhythmogenesis in associative networks.

The absence of a robust posterior dominant rhythm and the limited eyes-open/eyes-closed modulation are more consistent with impaired thalamo-cortical state regulation and reduced posterior network integrity than with demonstrable improvement in visual processing. Therefore, although the clinical observations included more consistent orientation and blink-mediated interaction, the qEEG findings do not justify inferring enhanced visual awareness from spontaneous rhythms alone.

Another interesting electrophysiological observation was the emergence of theta and slow-beta components in fronto-temporal areas in the follow-up. Conceptually, in severe encephalopathy, it can represent a partial re-engagement of cortico-subcortical loops, because pure delta dominance often accompanies profound disconnection and reduced network “bandwidth.” In this patient, the topography of the newly detectable rhythmogenesis—temporal and fronto-temporal—fits plausibly with the specific behavioral changes reported at follow-up, namely more synchronized affective responses (smiling and crying) and the appearance of a rudimentary communicative channel through eye blinking. These changes, however, should be interpreted as an exploratory electrophysiological observation. The temporal association between these qEEG changes and the modest behavioral changes is correlational only. In a single uncontrolled case, it is not possible to determine whether the electrophysiological differences reflect a direct treatment effect or a state variability during recording.

Mechanistic interpretation of MSC effects in chronic hypoxic-ischemic encephalopathy should explicitly separate biological plausibility from demonstrated mechanism. Across neurological indications, MSC activity is commonly attributed to paracrine immunomodulation, vascular-glial signaling, and trophic support of endogenous plasticity rather than neuronal replacement (Caplan, 2017; Galipeau and Sensébé, 2018). This mechanistic hypothesis is driven by pre-clinical and *in vitro* studies, and should not be interpreted as a proven explanation for the changes observed in this patient. Prior experimental work from our group provides a direct translational constraint: *in vitro*, adult human MSCs can be driven toward neuron-like phenotypes, yet they exhibit markedly limited electrophysiological maturation compared with fetal-derived neural precursors, with smaller inward Na+ currents and absent or immature firing behavior (Lepski et al., 2010a). *In vivo*, survival and apparent neuronal differentiation of MSCs depend strongly on microenvironmental permissivity (Lepski et al., 2010b), reinforcing conservative expectations that any functional effects in humans are more likely mediated through environmental reprogramming and circuit-level plasticity than through replacement of lost neurons.

Within that framework, the qEEG trajectory in this case is best described as persistent severe diffuse dysfunction with some qualitative longitudinal changes in band-power distribution and estimated source patterns that might indicate incipient network reorganization. Global low power and diffuse slowing persisted, and the limited eyes-open/eyes-closed reactivity remained abnormal. Accordingly, the electrophysiological findings should be considered hypothesis-generating biomarkers for future studies rather than evidence of clinical effectiveness or network restoration.

Besides the behavioral and electrophysiological changes reported herein, this report has its limitations. It describes a single uncontrolled compassionate-use case; therefore, therapeutic efficacy cannot be directly inferred. It should also be considered that disorders of consciousness may fluctuate independently of any intervention, even in chronic phases. Moreover, qEEG analysis was descriptive, and the LORETA provides estimated low-resolution source localization and should not be interpreted as precise anatomical mapping, particularly in the context of severe diffuse encephalopathy.

Recent regulated clinical studies continue to support the overall feasibility and short-term safety of intrathecal or intravenous MSC-based approaches in selected neurological contexts, while efficacy remains indication- and endpoint-dependent (Bydon et al., 2024; Harris et al., 2024; Yamaki et al., 2024; Schiess et al., 2025). Accordingly, the relevance of the present report lies in its description of a regulated, hypothesis-generating feasibility and safety case, which may help inform future prospective trials incorporating pre-registered endpoints, standardized consciousness measures, and harmonized electrophysiological pipelines.

## Data Availability

The raw data supporting the conclusions of this article will be made available by the authors, without undue reservation.
